# A Cross-Cultural and Trans-Generational Study: Links between Psychological Characteristics and Socio-Political Tendency amongst Urban Population in Afghanistan

**DOI:** 10.3390/ijerph18126372

**Published:** 2021-06-12

**Authors:** Hossein Kaviani, Sayed-Jafar Ahmadi

**Affiliations:** 1 Department of Psychology, University of East London (UEL), London E16 2RD, UK; 2 Counselling Department, Shaheed Prof. Rabbani Education, University Kabul, Kabul 1001, Afghanistan; sjahmadi2002@gmail.com

**Keywords:** personality, democratic values, political behaviour, culture, Afghanistan

## Abstract

Aim: This study examined how psychosocial characteristics might relate to adherence to democratic values among young and older people within two different cultural contexts in Afghanistan. Method: Self-report questionnaires were employed to measure empathy, theory of mind, gender role equality, openness to experiences, suggestibility, authoritarianism and support for democracy. A sample of 669 people from younger (18–25 years) and older (45 years and above) age groups from different cultural backgrounds in Afghanistan participated in the study. A series of MANOVAs were conducted to examine the cultural (Dari, Pashto), generational and gender differences on the study variables. Dari and Pashto speakers showed equal degrees of support for authoritarianism regardless of age difference. Results: The findings reveal that Dari speakers scored more highly on empathy, theory of mind, openness, gender role equality, democratic values and lower on suggestibility than Pashto speakers. Older Pashtun participants had lower scores on theory of mind than their younger counterparts. Hierarchical multiple regression analysis yields that gender role equality, openness and suggestibility predict support for democracy with gender role equality being the strongest predictor. Conclusion: The knowledge gained here would potentially be incorporated into the development of practical guidelines to be used by policy makers, education systems and the media to facilitate the process of democratization.

## 1. Introduction

After the Second World War, the Middle East went through immense geopolitical and regime changes, mostly as a result of foreign military and/or political interventions. More recently, this region underwent heavily planned radical actions carried out by US-led military forces that aimed to replace authoritarian regimes (e.g., in Afghanistan and Iraq) with a democratic political system. After more than a decade, both countries, more specifically Afghanistan, are still struggling to establish the foundations of democracy. With this in mind, the extent to which the foreign forces have accommodated any subtle psycho-social factors into their strategic plan of action in the region is open to question. Any research that is to examine this unattended area would lead to a potential, novel and innovative contribution. The present study aimed to identify some of the psycho-social variables (such as empathy, openness and suggestibility) that might underpin people’s political tendencies and behaviours and, more specifically, adherence to democratic values in an Afghan population. As age and culture have been found to have impact on political tendencies [1,2], the role of generational and cultural differences are also examined. In this study, education will also be examined and controlled for as it is deemed to have an essential influence on the support for democracy, with highly educated people being more supportive of democratic values [3].

Most of the research on the potential role of psycho-social characteristics in people’s political orientations have been mostly carried out in the Western countries, such as the USA, the United Kingdom and other European societies [4,5,6,7].

### 1.1. Psycho-Social Factors and Political Behaviour

Several psycho-social factors are examined as potential predictors of political tendency. These are empathy, theory of mind, gender role equality, openness to experience, suggestibility and authoritarianism. Empathy, as a personality factor and its cognitive component, theory of mind (perspective taking), are deemed to be pertinent to the adherence to democratic values [6]. The importance of distinguishing emotional from cognitive dimensions of empathy has, however, been emphasised [8]. Empathy is defined as people’s concern for others and how they feel, while theory of mind is considered the extent to which one might understand what other people think [8,9]. In the present study, these two constructs will be measured using independent scales. There is evidence that the ability to see the world from other people’s perspective can enhance qualities such as tolerance, tenderness, compassion and understanding [10,11]. Empathy is thought to enhance citizens’ positive attitudes towards out-group members that, in turn, can give rise to being more tolerant towards others’ different opinions [12]. Empathy, as a personality characteristic, would enhance the ability of overcoming biases and discrimination against the dissimilar group [13]. We have previously reported research findings which highlighted the different roles played by empathy and theory of mind in predicting political attitudes [14,15]. Such qualities may encourage more positive attitudes towards some aspects of democracy such as equality, impartial justice, freedom of speech and respect for the rights of minority groups [16,17]. In other words, people high on empathy are more likely to support democratic values. A study of adolescents supports this view [6], but little is known about the role played by empathy in the political attitudes in adults in general.

Another characteristic measured in this study is openness to new experiences. People high on openness are more receptive toward novel ideas, curious and imaginative, whilst those who are less open are more conservative, cautious and risk averse [18]. Openness might encourage flexibility and a greater tolerance of difference; that is to say, people high in openness are less likely to see political differences as threatening [17].

Egalitarian sex roles are another psycho-social factor that might have a pivotal link with support for democratic values. Research suggests that a belief in egalitarian sex roles would predict support for democracy [19,20]. In other words, belief in equality between men and women in family, workplace and social affairs resonates with the adherence to democratic values. It has been argued that differences in attitudes to gender equality is the main reason for the clash of values between the Islamic and the Western world [21]. As a marker of support for egalitarian values, perceptions of gender role equality may also resonate with adherence to democratic values [9,22]. Previous research on people from Middle Eastern countries supports the idea that egalitarian sex roles might reinforce the support for democracy [14,15]. Since distinct norms and roles are culturally indicated and socially prescribed for men and women in Middle Eastern countries, it could be a particularly pertinent variable to be examined in the present study. One of the factors which can foster positive attitudes towards gender role equality is education [23]; educated people are more likely to support egalitarian sex roles. As explained earlier, the education factor was controlled for in the present study.

Another factor that might discourage the development of democratic values is suggestibility. This is a personality trait which reflects the extent to which information is uncritically accepted by recipients [24]. That is, higher levels of suggestibility (that is, accepting information at face value) might result in lower levels of critical thinking that, in turn, could foster anti-democratic and authoritarian values [25]. This study also examines the effects of authoritarianism on readiness to embrace democratic values. People who score high on authoritarianism show a tendency towards conformity and are more likely to see civil liberties and democratic imperatives as a threat to the social order [26]. This is supported by a recent study of 51 nations that found valuing conformity, security and traditional values predicts right wing cultural attitudes, particularly among ideologically constrained nations [27].

### 1.2. Previous and Present Research

In two previous studies, the authors investigated how the differences in people’s socio-political attitudes and behaviours are underpinned by individual characteristics in samples from the Middle East compared with a sample from a Western country. The first study [14] examined the role played by psycho-social factors in predicting attitudes towards democratic values in Iranian and British people. The results provided support for the between-group differences on the psycho-social factors (such as empathy, theory of mind, gender role equality and openness) as well as evidence for the validity and reliability of the scales. The second study [15] examined the associations between the psycho-social characteristics and political tendencies across three groups; namely, new migrants, migrants with a bicultural background, and non-migrant citizens living in the UK. The results indicated that the bicultural group scored in between two other groups, which might be explained by learning over time through political socialization. 

The present study aimed to build on this earlier work by examining the potential links between the psycho-social factors that were previously found to underpin adherence to demographic values in a large sample from the general population of Afghanistan. It would not only offer a way of replicating the existing evidence, but also allow a more comprehensive understanding of the psycho-social foundations underpinning support for democracy in Afghanistan cultural context. The study examines the predictors of and attitudes towards democracy in different groups. Firstly, it examines the differences between the younger and older generations as well as different cultural groups (with a focus on Dari and Pashto speakers). Dari (a dialect of Farsi Language) and Pashto are both official languages of Afghanistan and are spoken by different cultural groups [28]. Pashto speakers are deemed mostly as those with Pashtun origin and Dari speakers as participants with other cultural backgrounds such as Tajik, Hazare and Qizilbash [29]. Language is seen as the most essential factor in the development and expression of culture, helping people to share and communicate values, views, traditions and feelings [30]. Afghanistan has a long history of cultural/ethnic clashes between Pashtuns and other ethnic groups to remain in political power. Over the past 200 years, the government has alternated between Pashtuns and their opposition with a mix of other cultural/ethnic groups, Tajiks and Qizilbashs in particular. The Taliban movement was the last attempt as such to revive a centralised Pashtun-dominated state in Afghanistan [31]. As culture plays such an important role in the political divide in Afghanistan, an examination of the effects of culture is supported in the present study. 

## 2. Method

### 2.1. Participants

This research was conducted using a convenience sample of the urban population in Kabul, Afghanistan. They were recruited from university students and office staff. The respondents (*n* = 700) were approached by five research assistants and invited to participate in the study, of which 669 agreed to do so. Sixty-five questionnaires were incomplete and were excluded from analysis. The sample included both Dari and Pashto speakers from young (18–25 years) and older (45 years and above) generations. Table 1 provides details of the completers. 

### 2.2. Procedure

The research complied with the British Psychological Society’s code of conduct [32] and was approved by the ethics committee of the first author’s university. The volunteers were given an informed consent form to read and sign. They were reassured that the participation in the study was optional and their details and data would be kept confidential. They were also advised that they would be free to withdraw from this study at any time. Data collection and management were coordinated by an onsite researcher. 

To enhance the quality of data collection, the research assistants were trained before being dispatched for recruitment. All research assistants were Afghan nationals and took part in two training workshops, which was coordinated via online communication and run by the principal researcher. The workshops aimed to help the research assistants facilitate the smooth and safe running of data collection. They were fully trained in observing safety and security regulations, spotting any potential risks and the importance of being aware of any cultural sensitivities. Bearing in mind the political situation in Afghanistan at the time, the importance of taking care to avoid harm or danger was emphasised. 

### 2.3. Measures

This study employed seven short-form questionnaires to assess the potential psycho-social predictors of adherence to democratic values, all validated in a previous study [14]. Unless otherwise indicated, higher scores on each of the variables represent higher levels of each of the constructs measured. 

Empathy: This is defined as one’s ability to comprehend the emotions and feelings of others. This measure includes 10 items, initially derived from Toronto Empathy Questionnaire (TEQ) [33]. The items (e.g., “I have tender, concerned feelings for people less fortunate than me”) were rated on a five-point scale ranging from 0 (never) to 4 (always). 

Theory of Mind (ToM): ToM encompasses six items (e.g., ‘‘When I am upset at someone, I usually try to ‘put myself in his shoes’ for a while’’) derived from Perspective Taking Sub-scale [34]. This measure assesses the cognitive component of empathy: i.e., one’s ability to understand others’ thoughts and viewpoints. To rate each item, a 5-point scale from 0 (‘does not describe me well’) to 4 (‘describes me well’) was used.

Openness to Experience: Openness, derived from the subscale of Neo-PI-R [35], includes 12 items (e.g., ‘’I have a lot of intellectual curiosity’’). One who is high in openness is particularly willing to engage in new activities, would consider new, perhaps unconventional ideas and holds belief in pluralistic values. Each item was rated using a 5-point scale from ‘strongly disagree’ (1) to ‘strongly agree’ (5). 

Gender Role Equality: This measure comprises of 10 items (e.g., “Domestic chores should be shared between husband and wife’’) of Egalitarian Sex Role Attitudes (ESRA) [36]. This scale assesses respondents’ beliefs and attitudes on role equality across gender. Each item was rated from 1 (‘strongly disagree’) to 5 (‘strongly agree’). 

Suggestibility: The seven item Multidimensional Iowa Suggestibility Scale (MISS) [24] was used to detect the extent to which one tends to accept and internalise inputs uncritically (e.g., “I am easily influenced by other people’s opinions”). A 5-point scale from 1 (‘not at all or very slightly’) to 5 (‘a lot’) was used to rate each item. 

Authoritarianism: Derived from Right Wing Authoritarianism (RWA) [37], this measure evaluates the tendency to follow conventionalism in both thinking and action. The scale consists of 9 items (e.g., ‘‘Our country needs a powerful leader, in order to destroy the radical and immoral currents prevailing in our society today’’) with response options ranging from 1 (‘strongly disagree’) to 4 (‘strongly agree’).

Adherence to Democratic Values: This scale includes 9 items (e.g., “Democracy may have its problems, but it’s better than other forms of government’’) from the scale Support for Democratic Values (SDV) [6] to examine to what extent people adhere to democratic values. Items were rated using a 4-point rating scale from 1 (‘strongly disagree’) to 4 (‘strongly agree’). 

### 2.4. Translation and Linguistic Adaptation of Questionnaires

Dari, Farsi and Pashto belong to the Indo-European language family. Dari can also be regarded as a dialect of Farsi (Persian) and is broadly spoken in different areas of Afghanistan as the primary language. Dari and Farsi can be deemed as analogous to British English and American English, by which people of both sides are able to communicate without any difficulty. Nonetheless, they are seen as different languages [10]. 

All questionnaires used had been translated (and culturally adapted) into Farsi and validated in two previous studies [14,15]. For the purposes of the present study, these measures were all adapted by a psycholinguist and a psychologist to be suitable for Dari speakers. After adaptation, they were given to five Dari speakers to check if the items were comprehended correctly. Some minor changes were made according to the feedback received. To provide a set of measures in Pashto, a translation–back translation procedure [38] was conducted. The measures were translated from Farsi to Pashto by two bilingual psychologists. Then, a bilingual professional translated them back to Farsi. Finally, the back translated questionnaires were compared with Farsi versions. Discrepancies were resolved through communication with translators and amendments were made accordingly. 

## 3. Results

### 3.1. Demographic Variations

Table 1 shows the demographic profile of the Dari and Pashto speaker samples in terms of age, gender, generation, culture and education. Concerning culture, the Dari speakers were mostly Tajik and Hazare, while the Pashto speakers, as expected, were Pashtun (95%). Other than this, the two samples are similar in age, gender, generation and education.

### 3.2. Between Group Differences

A series of three-way (Group (Dari, Pashto) × Generation (young, older) × Gender (male, female)) MANOVAs were conducted to detect the main and interaction effects for the variables (empathy, theory of mind, openness to experience, gender role equality, suggestibility, authoritarianism and democratic values), separately. As no gender effect was observed on any of these variables, it was excluded from subsequent analysis and a further series of two-way (Group (Dari, Pashto) × Generation (young, older)) MANOVAs were conducted to detect the main and interaction effects for the variables.

**Figure 1:**
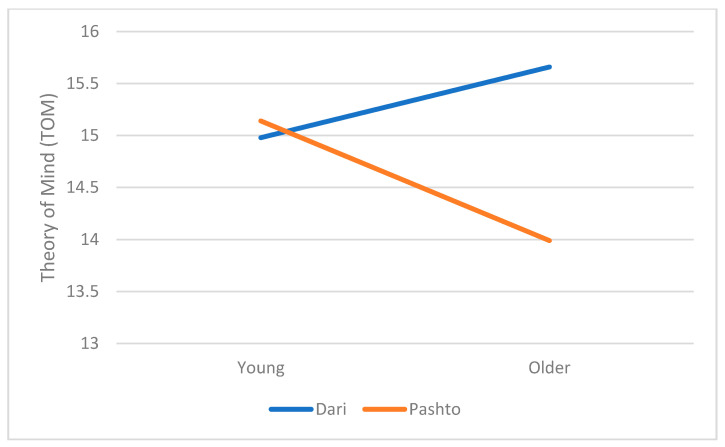
Theory of mind: A group × generation effect.

The MANOVA results, shown in Table 2, reveal that there is a group (Dari vs. Pashto speakers) main effect for all variables except authoritarianism. Specifically, Dari speakers tended to score more highly than Pashto speakers on empathy (Mean: 30.53 vs. 26.11, *p* < 0.001), theory of mind (15.32 vs. 14.58, *p* < 0.05), openness (35.78 vs. 33.04, *p* < 0.001) and gender role equality (37.22 vs. 34.46, *p* < 0.001). Pashto speakers were higher on suggestibility (Mean = 25.75) than their counterparts (mean = 23.28, *p* < 0.001). Both Dari and Pashto speakers scored equally on authoritarianism. Dari speakers also tended to have more favourable attitudes towards democratic values (24.70 vs. 22.49, *p* < 0.001). The main effects for generation were found on gender role equality and suggestibility. That is, younger people scored higher both on gender role equality (mean = 36.11) and suggestibility (mean = 25.07) than the older generation (mean = 34.35 and mean = 23.89, respectively). However, the only interaction effect observed was on theory of mind. This finding, together with the non-significant main generation effect, suggests that the older generation of Pashto speakers scored lower on theory of mind than their Dari counterparts (see Figure 1). Furthermore, t-tests proved no significant difference between the younger and older generations in the Dari speaker samples; however, the difference between young (Mean = 15.14) and older (mean = 13.99, *p* < 0.01) generations in the Pashto speaker samples appeared to be statistically significant (t292 = 2.78, *p* < 0.01).

### 3.3. Associations and Predictive Links

A hierarchical multiple regression (HMR) analysis was performed to examine the predictive value of each of the measures in accounting for attitudes towards democracy. To control for demographic variables (generation, gender, culture and education), they were introduced in step 1 and the psycho-social variables (empathy, theory of mind, openness, suggestibility, gender role equality and authoritarianism) were included in the second step. Table 3 summarizes details of the regressions for the study sample.

The demographic variables account for 13% of the variance in outcome variable (adherence to democratic values) (F (4, 603) = 21.90, *p* < 0.001) with culture factoring as the strongest, significant contributor (identified by betas). Controlling for the demographic variable, the psycho-social factors at step 2 turned out to explain 20% of the variations in the outcome variable (F (10, 604) = 13.50, *p* < 0.001), with gender role equality, openness, and suggestibility as the strongest predictive links, respectively (identified by betas).

In summary, a pronouncedly higher proportion of variance in democratic attitudes was explained by the psycho-social factors than the demographic variables. This is further supported by the intercorrelation results, shown in Table 4.

### 3.4. Intercorrelations between Psycho-Social Variables

Moreover, Table 4 demonstrates the Pearson correlation coefficients between the psycho-social variables. In the Dari speaker group, adherence to democratic values is positively correlated with openness and gender role equality, and negatively with suggestibility, whilst in the Pashto speaker group, adherence to democratic values is positively correlated with empathy and theory of mind. In both samples, negative correlations were observed between authoritarianism and empathy, theory of mind and openness. Moreover, a positive correlation was found, only in the Dari speaker samples, between authoritarianism and suggestibility. Gender role equality is positively correlated with empathy, theory of mind and openness in the Dari speaker group, and with empathy and theory of mind in the Pashto speaker group. Suggestibility is negatively correlated with openness in both samples and with gender role equality only in the Pashto speaker samples. As expected, empathy and theory of mind are positively correlated in both samples.

## 4. Discussion

The notion that personality characteristics might influence people’s socio-political tendencies has a long history in the social sciences [6,39]. The aim of the present study was to detect how a specific set of psycho-social variables would act as predisposing, subtle factors that may impede or promote the development of a democratic political system in Afghanistan. It also examined whether or not factors such as culture (Dari vs. Pashto speakers) and age (young vs. older generations) could play tangible roles in forming such attitudes. The findings reveal that Pashto speakers (almost all with a Pashtun cultural background) appeared to differ from Dari speakers (almost all with a non-Pashtun cultural background) on all variables except authoritarianism. More specifically, Dari speakers scored more highly on empathy, theory of mind, openness, gender role equality, democratic values and lower on suggestibility than their Pashto-speaker counterparts. Moreover, multiple regression analysis revealed that adherence to democratic values is mainly accounted for by some psycho-social factors, showing that gender role equality, openness and suggestibility (in a negative direction) were significant predictors of democratic values. The findings are further supported by intercorrelation results, which demonstrate that adherence to democratic values is positively correlated with openness and gender role equality, but negatively with suggestibility.

The above-mentioned factors were found to contribute to the support for democracy in previous research evidence [6,14,15]. The extent to which people adhere to equality, impartial justice, universal suffrage and freedom of expression would, by its very nature, distinguish between nondemocratic and democratic views [16,17]. Those high on empathy and its cognitive component, theory of mind, seem to be understanding, tolerant, tender, caring and compassionate in their relationships [10,11]. As a consequence, people who are empathic and are able to take the perspective of others would be particularly open to abide by democratic values such as freedom of speech, equality and respect for minority rights [9]. Support for equality, as a democratic value, certainly gives rise to a belief in gender equality [9,22]. This is in keeping with the finding on the link between gender role equality and adherence to democracy in the present study.

Gender role equality turned out to be the strongest predictor of adherence to democratic values. In addition, Dari speakers appeared to have stronger beliefs in gender role equality than their Pashtun counterparts. It is suggested that the belief in gender role equality is an essential factor that resonates with the adherence to domestic values [19], and can be deemed as the key indicator of the clash of values between the Muslim and the Western world [21]. This might signify that gender-related norms and roles are more culturally and socially defined and followed in the Pashto speaker group compared to their Dari speaker counterparts. One possible reason might have to do with the participants’ educational background. It is reported that educated individuals are more likely to hold more positive attitudes towards gender role equality [23] that, as a result, would boost adherence to democracy and undermine authoritarian attitudes. The education factor in the present study, however, turned out not to contribute to adherence to democratic values. This might suggest that there are additional factors in the Pashto speaker group that lead to a resistance to changing their gender role attitude. We would suggest that religiosity, as an integral part of a culture, could be a strong contributor. Religious traditions have the potential to both impede and/or facilitate tolerance, justice, compassion and peace [21]. Therefore, it can be argued that the religiosity elements that discourage the development of democracy should be explored. Pashtun people’s dedication to restrict traditions and cultural roles and rules compared to other ethnic groups in Afghanistan has been pointed out [40]. This pronounced difference along with above mentioned differences (on empathy, theory of mind, openness, gender role equality, democratic values and suggestibility) between the two ethnic/cultural groups (Pashtun and non-Pashtun) shows culture has a crucial part to play in how people might behave in a social or political contexts. It is in line with the assumption that psycho-social factors are culturally and socially constructed. One can emphasise that policy makers and those who are involved in the country’s strategy setting need to take into consideration these subtle culture-related behavioural patterns before coming to the implementation of any plan of action. However, caution should be taken as it seems to have given rise to some seemingly overstated stereotypes widely circulated by the media about this ethnic group suggesting they ‘have never known peace’ [41].

Openness to experience helps people be more receptive towards new ideas, and subsequently, be curious, perceptive and imaginative. It was anticipated that this would lead them to favour change and innovation over the conservative, cautious tradition [18]. Recent research found a positive relationship between openness and the willingness to extend political rights to disliked groups [42] which, in turn, led to the opposition to right-wing authoritarianism [43]. This could explain the linkage found in this study between openness and adherence to democratic values in Afghan participants that has also been found in previous studies [6,13,14,15]. The MANOVA results reveal that Pashtun speakers scored lower on openness to experiences and adherence to democracy. The reason for such a difference might be attributed to cultural differences between the two groups. It is suggested that Pashtuns tend to follow more traditionally and culturally defined rules and be more obedient towards religious decrees than other Afghan ethnic minorities [31]. This is further supported by a higher level of suggestibility among the Pashto speaker participants in this study.

Suggestibility was found to be an influential underpinning factor for the prediction of adherence to democracy in this study. Those high on suggestibility tend to accept information without engaging in critical thinking [24], possibly leading to rigidity in behaviour and attitude [44]. In theory, a person’s tendency for suggestibility should result in a preference for more conservative attitude and authoritarian values [25]. Moreover, in this study, suggestibility is inversely related to openness in both samples, and with gender role equality only in the Pashto speaker sample. With this in mind, it can be assumed that people with high levels of suggestibility, namely poor in critical thinking, are more likely to adhere to authoritarianism than values pertinent to democracy. The inverse relationship between suggestibility and adherence to democratic values were also found in two previous studies [14,15].

The only interaction effect obtained in the data analysis was between generation and theory of mind (TOM). More specifically, older participants in the Pashto-speaker sample had lower scores for theory of mind than their younger counterparts in their own group as well as the Dari speaker older generation subgroup. This might point to the fact that the younger generation taking part in this study regardless of their cultural background apparently enjoy nearly the same degree of ability to understand others’ perspectives and points of view. This TOM result is not consistent with the finding on empathy as neither a generational effect nor group–generation interaction was obtained for this variable. Empathetic concern is referred to as the extent to which people understand others’ feelings and sentiments, whilst TOM shows people’s ability to perceive others’ perspective [9]. In the present study, we used two different, more specific scales to measure these two components with this in mind that this might highlight a possible cleavage line between the cognitive and emotional components of empathy and their potentially different roles in adherence to democratic values.

Interestingly, both Dari and Pashto speakers showed equal degree of support for authoritarianism regardless of age difference. This is further supported by negative correlations between authoritarianism with empathy, theory of mind and openness in both groups. The inverse relationship between these variables and authoritarianism has been observed in previous studies [6,14,15]. The prevailing support for authoritarianism found in both groups in this study may highlight the fact that the general, dominant culture of Afghanistan fosters a conservative, authoritarian worldview among its citizens from different walks of life in this country [31]. It can, however, be argued that this finding contradicts the findings on the participants’ tendency (particularly observed in the younger generations and Dari speakers) towards democratic values. This discrepancy might be attributed to the socio-cultural transient period that Afghanistan has been experiencing over past decades [45], a limbo situation that is continuing at the time of writing.

The difference on authoritarianism across generation might be attributed to the so-called ‘information revolution era’ which has offered the young generation an exceptional opportunity to have access to a broad range of social media and internet-based communication interfaces [46]. This might, in its own right, expose the younger generation to a wide and diverse range of ideas and facilitate the recognition of differences and dissimilarities of others.

The question of importance in the present study would be whether or not the foreign forces intervened in countries such as Afghanistan, Iraq and Syria would have incorporated the subtlety of cultural and psycho-social properties into their strategic decisions and plan of action in the region. From this perspective, the present results seem to offer an innovative grounding that can be utilized by policy makers. Knowledge of the factors that underpin adherence to democracy can also inform educational strategies as well as a range of non-academic beneficiaries, including the media, non-governmental organisations (NGOs) and the public. The findings would also help policy makers gain a more comprehensive insight into the social and political behaviour of emerging democracies in the Middle East and their obstacles.

Overall, the results of the present study shed further light on the relationship between psycho-social factors including personality characteristics with political behaviour and tendency, and highlight cultural differences in this respect. However, the argument herein gains only partial significance owing to the limitations which should be addressed in future studies. First and foremost, the existing account rests on samples which are not fully representative of Afghanistan general population. Concerning this limitation, caution should be taken when generalizing the findings to the whole society. The underpinning factors examined in this study do not tick all the boxes in the potential list of related factors and variables. For certain, factors such as critical thinking and religiosity might contribute to this picture and should be added to the set of relevant variables in the hope of portraying a more comprehensive picture.

The experience and knowledge that we gained in this research and our previous studies [14,15] would potentially be incorporated into the development of a relevant education programme for school children at the national level. We are currently planning to conduct a study in Afghanistan to examine the impact of a ‘specifically designed training and education’ on the psycho-social characteristics such as empathy, theory of mind, compassion and tolerance that, in turn, would potentially influence people’s political tendencies and can eventually prepare the ground for a more peaceful society with positive cultural, political and economic consequences.

## Figures and Tables

**Table 1:** The sample demographic variations.

	Dari Speakers (*n* = 310)	Pashto Speakers (*n* = 294)
Gender		
Men	165 (53.2%)	148 (50.3%)
Women	145 (46.8%)	146 (49.7%)
Age		
Mean (SD)	35.83 (15.12)	37.47 (16.27)
Generation		
Younger	158 (51.0%)	146 (49.7%)
Older	145 (49.0%)	148 (50.3%)
Culture		
Tajik	104 (33.5%)	13 (4.5%)
Pashtun	18 (5.8%)	279 (94.9%)
Hazare	117 (37.5%)	1 (.3%)
Uzbek	10 (3.2%)	1 (.3%)
Others	61 (19.7%)	-
Education		
High School diploma	54 (17.4%)	73 (24.8%)
BSc	236 (76.1%)	203 (69.1%)
MSc and higher	20 (6.5%)	18 (6.1%)

**Table 2:** MANOVA results on target variables for group and generation factors and their interaction.

	Group (Dari–Pashto)	Generation (Young, Older)	Group × Generation
**Empathy**	F (1, 604) = 131.36 *p* < 0.001	F (1, 604) = 0.08 *Ns*	F (1, 604) = 0.10 *Ns*
**Theory of mind**	F (1, 604) = 6.53 *p* < 0.05	F (1, 604) = 0.66 *Ns*	F (1, 604) = 9.65 *p* < 0.01
**Openness**	F (1, 604) = 44.86 *p* < 0.001	F (1, 604) = 3.02 *Ns*	F (1, 604) = 1.54 *Ns*
**Gender role equality**	F (1, 604) = 30.34 *p* < 0.001	F (1, 604) = 6.23 *p* < 0.05	F (1, 604) = 1.67 *Ns*
**Suggestibility**	F (1, 604) = 34.48 *p* < 0.001	F (1, 604) = 8.04 *p* < 0.01	F (1, 604) = 1.32 *Ns*
**Authoritarianism**	F (1, 604) = 1.74 *Ns*	F (1, 604) = 2.71 *Ns*	F (1, 604) = 2.26 *Ns*
**Democratic values**	F (1, 604) = 82.86 *p* < 0.001	F (1, 604) = 1.78 *Ns*	F (1, 604) = 1.94 *Ns*

**Table 3:** Essential details of HMR.

	Βeta	R2 *change*
**Step1: Demographic**		0.13 **
Generation	0.01	
Gender	0.02	
Education	0.03	
Culture	0.34 **	
**Step 2: Psycho-social**		0.20 **
Empathy	0.07	
ToM	0.04	
Openness	0.13 *	
Gender role equality	0.15 **	
Suggestibility	−0.10 *	
Authoritarianism	0.02	

* *p* < 0.05, ** *p* < 0.01.

**Table 4:** Intercorrelations of variables in Dari and Pashtospeaker (**in bold**) samples.

	1	2	3	4	5	6	7
1. Empathy	1						
2. Theory of mind	0.17 ** 0.32 **	1					
3. Openness	0.12 * −0.09	0.02 **−0.04**	1				
4. Gender role equality	0.17 * 0.17 *	0.16 ** 0.19 **	0.20 ** 0.06	1			
5. Suggestibility	0.07 **−0.05**	−0.04 **−0.05**	−0.12 * −0.19 **	0.02 −0.25 **	1		
6. Authoritarianism	−0.18 ** −0.30 **	−0.12 * −0.23 **	−0.11 * −0.12 **	−0.05 **0.05**	0.12 * 0.06	1	
7. Democratic values	0.02 0.18 **	0.02 0.16 **	0.24 ** 0.10	0.23 *** 0.10	−0.15 ** −0.05	−0.03 **−0.08**	1

* *p* < 0.05, ** *p* < 0.01, *** *p* < 0.001

## Data Availability

The data presented in this study are available on request from the corresponding author. The data are not publicly available due to Security reason.

## References

[1] Muller E.N. (2013). Seligson, M.A. Civic Culture and Democracy: The Question of Causal Relationships. Am. Political Sci. Rev..

[2] Seo Y. (2017). Democracy in the ageing society: Quest for political equilibrium between generations. Futures.

[3] Evans G., Rose P. (2012). Understanding Education’s Influence on Support for Democracy in Sub-Saharan Africa. J. Dev. Stud..

[4] Angelo D., Dyson J. (1968). Personality and political orientation. Midwest J. Political Sci..

[5] Fatke M. (2017). Personality traits and political ideology: A first global assessment. Political Psychol..

[6] Miklikovaska M. (2012). Psychological underpinnings of democracy: Empathy, authoritarianism, self-steem, interpersonal trust, normative identity style, and openness to experience as predictors of support for democracy. Personal. Individ. Differ..

[7] Pratto F., Sidanius J., Stallworth L.M., Malle B.F. (1994). Social dominance orientation: A personality variable predicting social and political attitudes. J. Personal. Soc. Psychol..

[8] Preston S.D., de Waal F.B. (2002). Empathy: Its ultimate and proximate bases. Behav. Brain Sci..

[9] Raboteg-Saric Z., Hoffman M.L. (2001). Empathy and Moral Development: Implications for Caring and Justice. Contemp. Sociol. A J. Rev..

[10] Batson C.D., Polycarpou M.P., Harmon-Jones E., Imhoff H.J., Mitchener E.C., Bednar L.L., Klein T.R., Highberger L. (1997). Empathy and attitudes: Can feelings for a member of a stigmatized group improve feelings toward that group?. J. Personal. Soc. Psychol..

[11] Mikulincer M., Shaver P.R., Gillath O., Nitzberg R.E. (2005). Attachment, caregiving, and altruism: Boosting attachment security increases compassion and helping. J. Personal. Soc. Psychol..

[12] Telle N., Pfister H. (2012). Not only the miserable receive help: Empathy promotes prosocial behaviour toward the happy. Curr. Psychol..

[13] Finlay K.A., Stephan W.G. (2000). Improving Intergroup Relations: The Effects of Empathy on Racial Attitudes. J. Appl. Soc. Psychol..

[14] Kaviani H., Kinman G. (2017). Relationships between psychosocial characteristics and democratic values: A cross-cultural study. Res. Rev. J. Soc. Sci..

[15] Kaviani H., Kinman G., Salavati M. (2017). Bicultural Iranians’ political tendency: In between two cultures. J. Soc..

[16] Ikenberry G.J., Dahl R.A. (1999). On Democracy. Foreign Aff..

[17] Peffley M., Rohrschneider R. (2003). Democratization and political tolerance in seventeen countries: A multi-level model of democratic learning. Political Res. Q..

[18] McCrae R.R., Costa P.T. (2003). Personality in Adulthood: A Five-Factor Theory Perspective.

[19] Inglehart R., Norris P. (2003). Rising tide. Gender Equality and Cultural Change Around the World.

[20] Goodwin R. (2013). Personal Relationships Across Cultures.

[21] Norris P., Inglehart R. (2011). Sacred and Secular: Religion and Politics Worldwide.

[22] Kohlberg L. (1958). The Development of Modes of Moral Thinking and Choice in the Years 10 to 16. Ph.D. Dissertation.

[23] Shu X. (2004). Education and Gender Egalitarianism: The Case of China. Sociol. Educ..

[24] Kotov R.I., Bellman S.B., Watson D.B., Multidimensional Iowa Suggestibility Scale (MISS) (2004). Brief Manual. http://www.stonybrookmedicalcenter.org/system/files/MISS_FINAL_BLANK_0.pdf.

[25] Guyton E.M. (1988). Critical Thinking and Political Participation: Development and Assessment of a Causal Model. Theory Res. Soc. Educ..

[26] Cohrs J.C., Kielmann S., Maes J., Moschner B. (2005). Effects of right-wing authoritarianism and threat from terrorism on restriction of civil liberties. Anal. Soc. Issues Public Policy.

[27] Malka A., Soto C.J., Inzlicht M., Lelkes Y. (2014). Do needs for security and certainty predict cultural and economic conservatism? A cross-national analysis. J. Personal. Soc. Psychol..

[28] Brown K. (2005). Encyclopaedia of Language and Linguistics.

[29] Yarshater E.V.I., Sebeok T.A. (1971). Linguistics in South West Asia and North Africa. Current Trends in Linguistics.

[30] German F. (2006). Language and Ethnicity.

[31] Siddique A., Afghanistan’s Ethnic Divides (2012). CIDOB Policy Research Project. https://www.cidob.org/en/content/download/.../OK_ABUBAKAR%20SIDDIQUE.pdf.

[32] BPS (2009). Ethical Code of Conduct. http://www.bps.org.uk/sites/default/files/documents/code_of_ethics_and_conduct.pdf.

[33] Spreng R.N., McKinnon M.C., Mar R.A., Levine B. (2009). The Toronto Empathy Questionnaire: Scale development and initial validation of a factor-analytic solution to multiple empathy measures. J. Personal. Assess..

[34] Davis M.H. (1983). Measuring individual differences in empathy: Evidence for a multi- dimensional approach. J. Personal. Soc. Psychol..

[35] Costa P.T., McCrae R.R. (1992). Revised NEO Personality Inventory (NEO-PI-R) and NEO Five-Factor Inventory (NEO-FFI) Professional Manual.

[36] Suzuki A. (1991). Egalitarian sex role attitudes: Scale Development and Comparison of American and Japanese Women. Sex Roles.

[37] Zakrisson I. (2005). Construction of a short version of the Right-Wing Authoritarianism (RWA) scale. Personal. Individ. Differ..

[38] Brislin R.W. (1970). Back-translation for cross-cultural research. J. Cross-Cult. Psychol..

[39] Adorno T., Frenkel-Brunswick E., Levinson D., Sanford R. (1950). The Authoritarian Personality.

[40] Sungur Z.T. (2016). Early modern state formation in Afghanistan in relation to Pashtun Tribalism. Stud. Ethn. Natl..

[41] Khan A.R. (2015). The ultimate warriors: Why lasting peace will require not just talks with the Taliban, but rescuing the shattered Pashtun culture. Maclean’s.

[42] Oskarsson S., Widmalm S. (2016). Personality and Political Tolerance: Evidence from India and Pakistan. Political Stud..

[43] van Hiel A., Kossowska M., Mervielde I. (2000). The relationship between Openness to Experience and political ideology. Pers. Individ. Differ..

[44] Pires R., Silva D.R., Ferreira A.S. (2013). Personality styles and suggestibility: A differential approach. Pers. Individ. Differ..

[45] Suhrke A. (2007). Reconstruction as modernisation: The ‘post-conflict’ project in Afghanistan. Third World Q..

[46] Yun S., Chang W.Y. (2011). Political participation of teenagers in the information era. Soc. Sci. Comput. Rev..

